# Supplementary material for: Possible depth-resolved reconstruction of shear moduli in the cornea following collagen crosslinking (CXL) with optical coherence tomography and elastography
Source: ArXiv. 2023 Jun 26:arXiv:2306.15018v1. Preprint. [Version 1] (PMC10327230)
Supplement: 1 [file NIHPP2306.15018V1-supplement-1.pdf]

Supplementary Material:

Possible depth-resolved reconstruction of shear moduli in the cornea following collagen crosslinking (CXL) with optical coherence tomography and elastography.

GABRIEL REGNAULT,[1*] MITCHELL A. KIRBY,[1] RUIKANG K. WANG[1,2], TUENG T. SHEN,[2,3] MATTHEW O'DONNELL[1] AND IVAN PELIVANOV[1]

[1]*Department of Bioengineering, University of Washington, Seattle, USA.*
[2]*Department of Ophthalmology, University of Washington, Seattle, USA.*
[3]*School of Medicine, University of Washington, Seattle, USA.*
**gregnaul@uw.edu*

Supplementary Material 1:

Guided mechanical wave propagation in a multilayered NITI material

Assume that a CXL-treated cornea can be modelled as a laminate of NITI layers. Each layer has a finite thickness with defined in- and out-of-plane elastic moduli. The first layer is bounded on the top by air and the last layer is bounded on the bottom by a liquid to mimic corneal *in vivo* conditions. Because collagen fibers are oriented randomly in the equatorial plane, it possesses symmetry across fibers, *i.e.* in the direction normal to the corneal surface, which is mathematically described as transverse isotropy.

1.1 Dimensionless equations of motion in a multilayered NITI material

Because the macroscopic corneal symmetry did not change following crosslinking, we assume that it contains at least two layers of finite thickness with distinct in- and out-of-plane shear moduli μ and G respectively. The density of every layer, ρ, is assumed to be identical for all layers and equal to that of the liquid bounding the lower layer, $\rho_l = \rho = 1000$ kg·m^{-3}. Using Voigt's notation, the stress-strain relationship in each layer can be written as:

$$\begin{bmatrix}\sigma_{xx}\\ \sigma_{yy}\\ \sigma_{zz}\\ \tau_{yz}\\ \tau_{xz}\\ \tau_{xy}\end{bmatrix} = \begin{bmatrix}\lambda+2\mu & \lambda & \lambda & & & \\ \lambda & \lambda+2\mu & \lambda & & & \\ \lambda & \lambda & \lambda+\delta & & & \\ & & & G & & \\ & & & & G & \\ & & & & & \mu\end{bmatrix}\begin{bmatrix}\epsilon_{xx}\\ \epsilon_{yy}\\ \epsilon_{zz}\\ \gamma_{yz}\\ \gamma_{xz}\\ \gamma_{xy}\end{bmatrix}, \qquad \text{(S1)}$$

where $\lambda = \rho c_p^2 - 2\mu$ is the Lamé coefficient, and c_p is the speed of the longitudinal wave that ensures incompressibility of the material (the Poisson's ratio of each layer taken individually is $\nu \sim 0.5$).

Newton's second law yields the wave equation of motion in terms of the displacement vector $\vec{u} = (u, v, w)$,

$$\frac{\partial \sigma_{ij}}{\partial x_j} = \rho \frac{\partial u_i}{\partial t^2}. \qquad \text{(S2)}$$

Because our OCE experiments use AμT acting as a pseudo-line source, we can assume a plane-strain state (no displacement polarized along the y-axis: $v = 0$, and no propagation along the y-axis: $u = u(x, z), w = w(x, z)$). As such, in the NITI model, the equations of motion can be expressed as:

$$\rho u_{tt} = (\lambda + 2\mu)u_{xx} + Gu_{zz} + (\lambda + G)w_{xz} , \quad \text{(S3)}$$
$$\rho w_{tt} = Gw_{xx} + (\lambda + 2\mu)w_{zz} + (\lambda + G)u_{xz} , \quad \text{(S4)}$$

where the lower indexes indicate derivatives with time (t) or spatial coordinates (x, z).

By introducing the scales:

Position: $x \sim h = \sum_i h_i$,
Displacement: $u \sim h$,
Time: $t \sim h \cdot \sqrt{\frac{\mu_M}{\rho}}$,
Frequency: $f \sim \sqrt{\frac{\mu_M}{\rho}} \cdot \frac{1}{h}$,
Wavenumber: $k \sim \frac{1}{h}$,

where h relates to the overall thickness, h_i to the thickness of thei_{th} layer , and $\mu_M = \max(\mu_i)$ is the maximum in-plane shear modulus among the layers. We can then define the dimensionless parameters $t^* = \frac{t}{h}\sqrt{\frac{\mu_M}{\rho}}$, $u^* = \frac{u}{h}$, $x^* = \frac{x}{h}$, $f^* = fh\sqrt{\frac{\rho}{\mu_M}}$, $k^* = kh$.

By applying this change of variables, we have:

$$\frac{\partial u}{\partial x} = \frac{\partial h \times u^*}{\partial x^*} \times \frac{\partial x^*}{\partial x} = h \cdot \frac{\partial u^*}{\partial x^*} \cdot \frac{1}{h} = \frac{\partial u^*}{\partial x^*} , \quad \text{(S5)}$$
$$\frac{\partial^2 u}{\partial x^2} = \frac{\partial}{\partial x}\left(\frac{\partial u^*}{\partial x^*}\right) = \frac{1}{h} \cdot \frac{\partial^2 u^*}{\partial x^{*2}} , \quad \text{(S6)}$$
$$\frac{\partial^2 u}{\partial t^2} = \frac{\mu_M}{\rho h} \cdot \frac{\partial^2 u^*}{\partial t^{*2}} , \quad \text{(S7)}$$

which eventually leads to the dimensionless equations of motion:

$$\rho . u_{tt}^* \cdot \frac{\mu_M}{\rho h} = (\lambda + 2\mu)u_{xx}^* \cdot \frac{1}{h} + Gu_{zz}^* \cdot \frac{1}{h} + (\lambda + G)w_{xz}^* \cdot \frac{1}{h}, \quad \text{(S8)}$$
$$\rho\mu_M \cdot w_{tt}^* \frac{\mu_M}{\rho h} = Gw_{xx}^* \cdot \frac{1}{h} + (\lambda + 2\mu)w_{zz}^* \cdot \frac{1}{h} + (\lambda + G)u_{xz}^* \cdot \frac{1}{h}, \quad \text{(S9)}$$

and after rearranging:

$$u_{tt}^* = \beta^2 u_{xx}^* + \alpha^2 u_{zz}^* + \gamma^2 w_{xz}^* , \quad \text{(S10)}$$
$$w_{tt}^* = \alpha^2 w_{xx}^* + \beta^2 w_{zz}^* + \gamma^2 u_{xz}^* , \quad \text{(S11)}$$

with

$$\alpha^2 = \frac{G}{\mu_M} , \quad \text{(S12)}$$
$$\beta^2 = \frac{(\lambda+2\mu)}{\mu_M} , \quad \text{(S13)}$$
$$\gamma^2 = \frac{(\lambda+G)}{\mu_M} . \quad \text{(S14)}$$

For the sake of simplicity, we will later omit the asterisk '*' symbol to refer to dimensionless variables.

1.2 Dispersion relationship for guided mechanical waves in a multilayered NITI material

Consider here a material containing multiple layers with identical density, each of finite thickness h_i. As such, there is a system of $[2 \times N]$ equations for the N layers:

$$u_{i,tt} = \beta_i^2 u_{xx} + \alpha_i^2 u_{zz} + \gamma_i^2 w_{xz}\ , \quad \text{(S15)}$$
$$w_{i,tt} = \alpha_i^2 w_{xx} + \beta_i^2 w_{zz} + \gamma_i^2 u_{xz}\ , \quad \text{(S16)}$$
$$\alpha_i^2 = \frac{G_i}{\mu_N}, \quad \text{(S17)}$$
$$\beta_i^2 = \frac{(\lambda_i + 2\mu_i)}{\mu_N}, \quad \text{(S18)}$$
$$\gamma_i^2 = \frac{(\lambda_i + G_i)}{\mu_N}. \quad \text{(S19)}$$

Assume harmonic solutions of the form:

$$u_i(x,z,t) = A_i e^{i(kx + l_i z - \omega t)}\ , \quad \text{(S20)}$$
$$w_i(x,z,t) = B_i e^{i(kx + l_i z - \omega t)}\ . \quad \text{(S21)}$$

Without loss of generality, assume $B_i = 1$. By substituting equations (S20) and (S21) into the equations of motion (S15) and (S16), the constants l_i and A_i can be determined for each frequency and wavenumber such that:

$$l_i = \pm\sqrt{\frac{1}{2}\left[\phi_\mathrm{i} \pm \sqrt{\phi_i - 4q_{\alpha_i}^2 q_{\beta_i}^2}\right]}\ , \quad \text{(S22)}$$

$$A_i = \pm\left[-\frac{\sqrt{2}\frac{\gamma_i^2 k}{\alpha_i^2}\sqrt{\phi_i \pm \sqrt{\phi_i - 4q_{\alpha_i}^2 q_{\beta_i}^2}}}{\phi_i + \frac{2\beta_i^2}{\alpha_i^2}q_{\beta_i}^2 \pm \sqrt{\phi_i - 4q_{\alpha_i}^2 q_{\beta_i}^2}}\right], \quad \text{(S23)}$$

where

$$\phi_i = \frac{\gamma_i^4 k^2}{\alpha_i^2 \beta_i^2} - \frac{\alpha_i^2}{\beta_i^2} q_{\alpha_i}^2 - \frac{\beta_i^2}{\alpha_i^2} q_{\beta_i}^2\ , \quad \text{(S24)}$$
$$q_{\alpha_i}^2 = k^2 - \frac{\omega^2}{\alpha_i^2}, \quad \text{(S25)}$$
$$q_{\beta_i}^2 = k^2 - \frac{\omega^2}{\beta_i^2}. \quad \text{(S26)}$$

The full solutions for a given layer can be expressed as the combination of 4 partial waves:

$$u_i(x,z,t) = \sum_{j=1}^{4} C_{i,j} A_{i,j} e^{il_{i,j} z} e^{i(kx - \omega t)}\ , \quad \text{(S27)}$$
$$w_i(x,z,t) = \sum_{j=1}^{4} C_{i,j} e^{il_{i,j} z} e^{i(kx - \omega\ )}\ . \quad \text{(S28)}$$

In the fluid, the dimensionless velocity potential is:

$$\Phi = C_{N,5} e^{\epsilon z} e^{i(kx - \omega t)}\ , \quad \text{(S29)}$$

where $\epsilon = \sqrt{k^2 - \frac{\omega^2}{\delta^2}}$ and $\delta^2 = \frac{\rho}{\mu_N} c_p^2$.

The constants $C_{i,j}$ are chosen so that the solutions satisfy the boundary conditions. Traction free air-solid interface sets

$$\sigma_{1,xz} = 0 \quad \text{at} \quad z_1 = 1\ , \quad \text{(S30)}$$
$$\sigma_{1,zz} = 0 \quad \text{at} \quad z_1 = 1\ . \quad \text{(S31)}$$

Continuity of normal components of stress and displacement between each layer give

$$\sigma_{i,xz} = \sigma_{i+1,xz} \qquad \text{at} \qquad z_i = 1 - \frac{\sum_{k=1}^{i} h_k}{h}\,, \tag{S32}$$

$$\sigma_{i,zz} = \sigma_{i+1,zz} \qquad \text{at} \qquad z_i = 1 - \frac{\sum_{k=1}^{i} h_k}{h}\,, \tag{S33}$$

$$u_i = u_{i+1} \qquad \text{at} \qquad z_i = 1 - \frac{\sum_{k=1}^{i} h_k}{h}\,, \tag{S34}$$

$$w_i = v_{i+1} \qquad \text{at} \qquad z_i = 1 - \frac{\sum_{k=1}^{i} h_k}{h}\,. \tag{S35}$$

Medium-fluid boundary conditions (zero tangential stress, continuity of normal stress components and speed) set

$$\sigma_{N,xz} = 0 \qquad \text{at} \qquad z_N = 0\,, \tag{S36}$$

$$\sigma_{N,zz} = \sigma_{zz}^{f} \qquad \text{at} \qquad z_N = 0\,, \tag{S37}$$

$$\dot{w}_N = \dot{w}^{f} \qquad \text{at} \qquad z_N = 0\,. \tag{S38}$$

Substituting the general solution into the boundary conditions yields a [4N+1 × 4N+1] homogeneous system for the coefficient: $\mathbf{Mc} = \mathbf{0}$. This system has a nontrivial solution if and only if the determinant of **M** (see Eq. (S39) below) is zero. For a given angular frequency ω, the wavenumber k associated with different wave types (pure shear or guided modes) can be found by minimizing the absolute value of the determinant:

$$M = \left[\begin{matrix}
(l_{1,1}A_{1,1} + k)e^{il_{1,1}} & (l_{1,2}A_{1,2} + k)e^{il_{1,2}} & (l_{1,3}A_{1,3} + k)e^{il_{1,3}} & (l_{1,4}A_{1,4} + k)e^{il_{1,4}} \\
[k(\gamma_1^2 - \alpha_1^2)A_{1,1} + \beta_1^2 l_{1,1}]e^{il_{1,1}} & k(\gamma_1^2 - \alpha_1^2)A_{1,2} + \beta_1^2 l_{1,2}]e^{il_{1,2}} & k(\gamma_1^2 - \alpha_1^2)A_{1,3} + \beta_1^2 l_{1,3}]e^{il_{1,3}} & k(\gamma_1^2 - \alpha_1^2)A_{1,4} + \beta_1^2 l_{1,4}]e^{il_{1,4}} \\
(l_{1,1}A_{1,1} + k)e^{il_{1,1}z_2} & (l_{1,2}A_{1,2} + k)e^{il_{1,2}z_2} & (l_{1,3}A_{1,3} + k)e^{il_{1,3}z_2} & (l_{1,4}A_{1,4} + k)e^{il_{1,4}z_2} \\
[k(\gamma_1^2 - \alpha_1^2)A_{1,1} + \beta_1^2 l_{1,1}]e^{il_{1,1}z_2} & k(\gamma_1^2 - \alpha_1^2)A_{1,2} + \beta_1^2 l_{1,2}]e^{il_{1,2}z_2} & k(\gamma_1^2 - \alpha_1^2)A_{1,3} + \beta_1^2 l_{1,3}]e^{il_{1,3}z_2} & k(\gamma_1^2 - \alpha_1^2)A_{1,4} + \beta_1^2 l_{1,4}]e^{il_{1,4}z_2} \\
A_{1,1}e^{il_{1,1}z_2} & A_{1,2}e^{il_{1,2}z_2} & A_{1,3}e^{il_{1,3}z_2} & A_{1,4}e^{il_{1,4}z_2} \\
e^{il_{1,1}z_2} & e^{il_{1,2}z_2} & e^{il_{1,3}z_2} & e^{il_{1,4}z_2} \\
0 & 0 & 0 & 0 \\
\vdots & \vdots & \vdots & \vdots
\end{matrix}\right.$$

$$\begin{matrix}
0 & 0 & 0 & 0 & \cdots \\
0 & 0 & 0 & 0 & \cdots \\
-(l_{2,1}A_{2,1} + k)e^{il_{2,1}z_2} & -(l_{2,2}A_{2,2} + k)e^{il_{2,2}z_2} & -(l_{2,3}A_{2,3} + k)e^{il_{2,3}z_2} & -(l_{2,4}A_{2,4} + k)e^{il_{2,4}z_2} & \cdots \\
-[k(\gamma_2^2 - \alpha_2^2)A_{2,1} + \beta_2^2 l_{2,1}]e^{il_{2,1}z_2} & -[k(\gamma_2^2 - \alpha_2^2)A_{2,2} + \beta_2^2 l_{2,2}]e^{il_{2,2}z_2} & -[k(\gamma_2^2 - \alpha_2^2)A_{2,3} + \beta_2^2 l_{2,3}]e^{il_{2,3}z_2} & -[k(\gamma_2^2 - \alpha_2^2)A_{2,4} + \beta_2^2 l_{2,4}]e^{il_{2,4}z_2} & \cdots \\
-A_{2,1}e^{il_{2,1}z_2} & -A_{2,2}e^{il_{2,2}z_2} & -A_{2,3}e^{il_{2,3}z_2} & -A_{2,4}e^{il_{2,4}z_2} & \cdots \\
-e^{il_{2,1}z_2} & -e^{il_{2,2}z_2} & -e^{il_{2,3}z_2} & -e^{il_{2,4}z_2} & \cdots \\
0 & 0 & 0 & 0 & \cdots \\
\vdots & \vdots & \vdots & \vdots & \vdots
\end{matrix}$$

$$\left.\begin{matrix}
0 & 0 & 0 & 0 & 0 \\
\vdots & \vdots & \vdots & \vdots & \vdots \\
l_{N,1}A_{N,1} + k & l_{N,2}A_{N,2} + k & l_{N,3}A_{N,3} + k & l_{N,4}A_{N,4} + k & 0 \\
k(\gamma_N^2 - \alpha_N^2)A_{N,1} + \beta_N^2 l_{N,1} & k(\gamma_N^2 - \alpha_N^2)A_{N,2} + \beta_N^2 l_{N,2} & k(\gamma_N^2 - \alpha_N^2)A_{N,3} + \beta_N^2 l_{N,3} & k(\gamma_N^2 - \alpha_N^2)A_{N,4} + \beta_N^2 l_{N,4} & \frac{\omega\rho^f}{\rho} \\
\omega & \omega & \omega & \omega & -i\epsilon
\end{matrix}\right]. \tag{S39}$$

Supplementary Material 2:

Effective A_0 guided mode for a multilayered NITI medium

2.1 Do engineering moduli correctly define the effective guided mechanical wave in a multi-layered NITI medium?

As discussed in Section 3.3 of the main manuscript, elastic moduli determined for all layers of CXL-treated cornea can be used to compute effective corneal engineering moduli, where μ_{eff} uses a simple mixture rule (Eq. (5) of the main manuscript) whereas G_{eff} requires the inverse mixture rule (Eq. (4) of the main manuscript).

We also checked whether this model can describe guided wave behavior in the partially crosslinked cornea when considered as an effective homogeneous material. First, we applied the fitting procedure to the treated cornea, considering it as a single layer with 'effective' moduli (see Fig. S1). We could determine a pair of moduli for the computed *k-f* spectrum that best fit the A_0-mode. These effective 'guided wave' elastic moduli were $G_{guided} = 127.5 \mp (12, 17)$ kPa and $\mu_{guided} = 9.3 \mp (8, 18)$ MPa, with a 0.953 goodness of fit.

These values clearly do not equal the corneal effective engineering moduli, $G_{eff} = 77.3 \mp (6{,}10)$ kPa and $\mu_{eff} = 15.4 \mp (8{,}11)$ MPa, computed with the mixture rules described by Eqs. (4), (5) using moduli measured with OCE in both layers. That is, it appears that the effective guided wave behavior in a multi-layered NITI medium is not described by the corneal effective engineering moduli which determine its low-frequency quasi-static deformation.

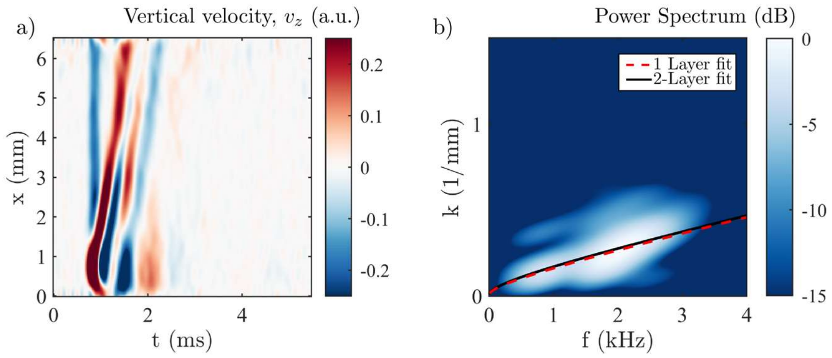

Figure S1. Fitting experimental results obtained for a partially (in-depth) CXL-treated cornea with either 1- or 2-layer analytical model. a) Measured vertically polarized top-surface vibration velocity field (*x-t* plot) of the guided wave. Its 2D-FFT spectrum with best-fit dispersion curves for 1-layer (red dashed line) and 2-layer (black solid line) models superimposed.

2.2 A single-layer approximation for the computation the guided mechanical wave dispersion in a multi-layered NITI medium

Using our analytical model, we further investigated the accuracy of the mixture rule (Eqs. (4) and (5)) to predict the effective 'guided wave' behavior. We considered different distributions of stiffness: i) a two-layer case, as assumed for CXL-treated corneas; ii) a five-layer case with random distribution of stiffness and thickness of the layers; iii) a medium with both G and μ following an exponential decay in stiffness from top to bottom. In all cases, the total medium thickness was h=500 μm and its top and bottom layers replicated the corneal boundary

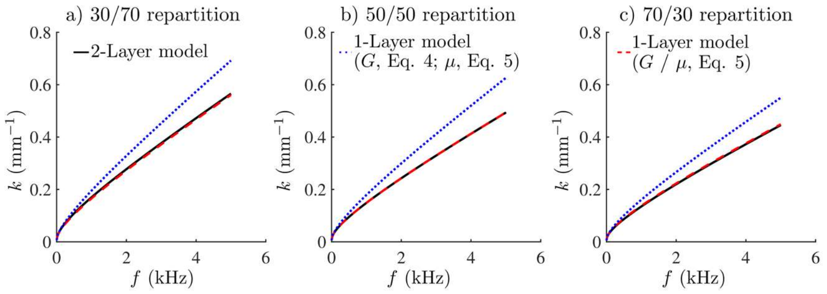

Figure S2. Computation of A_0-mode dispersion spectrum using different models and sets of mechanical moduli. In all 3 panels, black solid line corresponds to the exact solution obtained with the 2-layer model; blue dotted line corresponds to a 1-layer model with effective engineering mechanical moduli (Eq. 4 for G and Eq. 5 for μ); red dashed line corresponds to a 1-layer model with both effective moduli computed with Eq. 5.
Three different thicknesses of the top layer are considered a) 150 μm (30/70 repartition), b) 250 μm (50/50 repartition), and c) 350 μm (70/30 repartition). Moduli of the top layer are G_{ant} = 300 kPa and μ_{ant} = 30 MPa and for the bottom layer are G_{pos} = 60 kPa and μ_{pos} = 5 MPa, and the total thickness is h = 500 μm for all 3 cases.

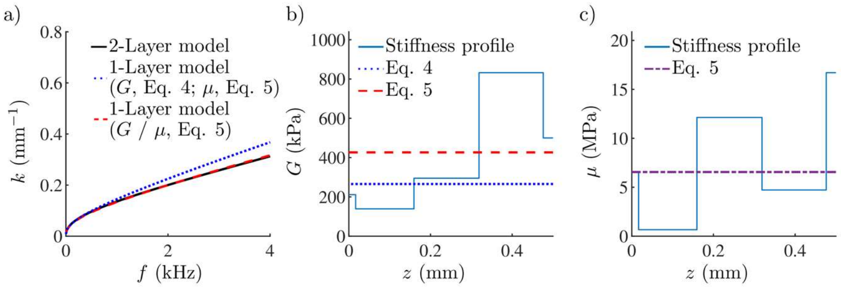

Figure S3. Computation of A_0-mode dispersion spectrum in a 5-layer medium using different models and sets of mechanical moduli. In panel a), black solid line corresponds to the exact solution obtained with the 2-layer model; blue dotted line corresponds to a 1-layer model with effective engineering mechanical moduli (Eq. 4 for G and Eq. 5 for μ); red dashed line corresponds to a 1-layer model with both effective moduli computed with Eq. 5. b) Distribution of modulus G in the 5-layer medium (light blue solid line), and its effective value obtained with Eq.4 (blue dotted line) and with Eq.5 (red dashed line). c) Distribution of modulus μ in the 5-layer medium (light blue solid line), and its effective value obtained with Eq.5 (purple dash-dotted line). Total thickness of the medium is h = 500 μm.

conditions. The Matlab scripts to reproduce these results are provided in Supplementary Material.

Results for case i) are presented in Fig. S2 for a material containing two layers with G_{ant} = 300 kPa and μ_{ant} = 30 MPa, and G_{pos} = 60 kPa and μ_{pos} = 5 MPa. For this case, we also explore different ratios between thicknesses of anterior and posterior layers. Results show that the A_0-mode computed with the effective engineering moduli using the mixture rules of Eqs. (4) and (5) does not match the exact analytical solution computed using the individual stiffness moduli of the layers, *i.e.*, using the 2-layered model directly. The difference is especially pronounced in the high-frequency range.

Results for case ii) are presented in Fig. S3. The distribution of stiffness for respectively G and μ are shown in Figs. S3 b,c). The thicknesses of layers were random with the total thickness of h = 500 μm. As for case i), we also see that assuming a single layer material with averaged moduli computed using engineering effective mechanical moduli does not accurately predict A_0-mode dispersion.

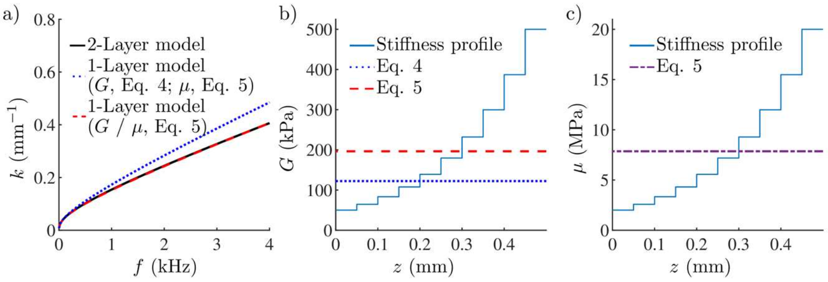

Figure S4. Computation of A_0-mode dispersion spectrum in a 10-layer medium with an exponential decay of elastic moduli using different models and sets of mechanical moduli. In panel a), black solid line corresponds to the exact solution obtained with the 2-layer model; blue dotted line corresponds to a 1-layer model with effective engineering mechanical moduli (Eq. 4 for G and Eq. 5 for μ); red dashed line corresponds to a 1-layer model with both effective moduli computed with Eq. 5. b) Distribution of modulus G in the 5-layer medium (light blue solid line), and its effective value obtained with Eq.4 (blue dotted line) and with Eq.5 (purple dash-dotted line). c) Distribution of modulus μ in the 5-layer medium (light blue solid line), and its effective value obtained with Eq.5 (red dashed line). Total thickness of the medium is $h = 500$ μm.

Results for case iii) are presented in Fig. S4. The distribution of stiffness for respectively G and μ are shown in Figs. S4 b, c). We considered 10 layers where G decreased exponentially from 500 kPa on top to 50 kPa at the bottom and μ decreased exponentially as well from 20 MPa on top to 2 MPa at the bottom. As for both cases i) and ii), the effective mechanical moduli do not describe the dispersion curve accurately, especially over the high-frequency range.

Thus, we can conclude that the mixture rules providing effective engineering moduli of multi-layered NITI media do not accurately describe guided wave behavior. As such, reconstruction of effective moduli from OCE measurements in a partially CXL-treated cornea should be performed with care.

The second question is which mixture model would describe guided wave behavior in a partially CXL-treated cornea with reasonable accuracy. This is an open and non-trivial question outside the scope of this paper. However, as shown in Figs. S2, S3 and S4, a simple direct mixture rule for both in- and out-of-plane moduli (Eq. (5)) produces a dispersion curve closely matching the N-layer model.

For the two-layer case (Fig. S2), equal thickness layers (50/50 split ratio, Fig. S2b) show a near-exact match, while the larger the difference between the layer thicknesses, the larger the difference between the solutions, although this difference remains small. In Fig. S3 (random thickness distribution), the simple mixing rule provides a reasonably good match, even though there is a small difference in the dispersion curves. In Fig. S4 (exponential decay of moduli), there is an almost exact match between the single layer effective model and the N-layer case.

Based on this observation, we assume that a simple mixture rule applied to both mechanical moduli μ and G provides a reasonable description of guided wave dispersion in multi-layer NITI materials. The effective guided model and the exact N-layer solution only differ slightly for an unequal distribution of thicknesses. However, we note that this rule is empirical and is not absolutely accurate. Finding an exact analytical solution may be difficult and include frequency-dependent terms. This is a subject for future studies.
